# Relationships between multivitamins, blood biochemistry markers, and BMC and BMD based on RF: A cross-sectional and population-based study of NHANES, 2017–2018

**DOI:** 10.1371/journal.pone.0309524

**Published:** 2025-01-29

**Authors:** Lijuan Xu, Mengqi Wu, Ying Zhang, Hongsheng Kun, Jiangbao Xu

**Affiliations:** 1 Jianshan People’s Hospital, Jiaxing, Zhejiang Province, China; 2 Center for Reproductive Medicine, Department of Pediatrics, Zhejiang Provincial People’s Hospital, Xiacheng, Hangzhou, P.R. China; 3 The Quzhou Affiliated Hospital of Wenzhou Medical University, Quzhou People’s Hospital, Quzhou, China; Universidade de Trás-os-Montes e Alto Douro: Universidade de Tras-os-Montes e Alto Douro, PORTUGAL

## Abstract

**Background:**

Previous studies have separately suggested a possible association between the vitamin exposure, blood biochemical indicators, and bone density. Our study aimed to investigate the relationship between vitamin exposure serum concentrations, blood biochemical indicator serum concentrations, and **BMC** and **BMD** using the **NHANES** 2017–2018 nutrient survey data. This population-based cross-sectional study aimed to explore these associations.

**Methods:**

In this study, we measured vitamin serum concentrations, serum ion serum concentrations, and serum biochemical indicators in adults participating in the **NHANES**. Skeletal status was assessed by evaluating **BMC** and **BMD** in the whole body. Given the inclusion of multiple variables and diverse data types, we used the **RF** to fit a multivariable model to estimate the associations between vitamin serum concentrations, blood biochemical indicator serum concentrations, and skeletal status.

**Results:**

Under the dimension reduction and comparison selection of **RF** model, we identified **ALP**, **CPK**, and creatinine serum concentrations as the most important factors associated with **BMC** and **BMD** in multiple skeletal sites, and the gender, age, height, weight, and body mass index which were found to be related to **BMC** and **BMD** in different skeletal sites. Vitamin D and blood calcium serum concentrations were not the important factors associated with **BMC** and **BMD** and the three blood biochemical indexes were more important than the vitamin level for **BMC** and **BMD**.

**Conclusion:**

The effect of vitamin serum concentrations and blood calcium serum concentrations on human bone density was not significant. **ALP**, **CPK** and creatinine serum concentrations body development indicators were identified as the most important factors related to bone status. The **RF** model can be used to comprehensively evaluate the effects of vitamin content and blood biochemistry serum concentrations in adults on **BMC** and **BMD.**

## 1. Introduction

The health of the skeletal system is a major public health concern in modern medicine [1]. Bone mass is commonly used to evaluate skeletal status, and bone mineral content (**BMC**) and bone mineral density (**BMD**), measured using techniques such as dual-energy X-ray, quantitative ultrasound, computed tomography, or magnetic resonance imaging, are reliable indicators of bone mass [2, 3]. **BMC** and **BMD** have long been the focus of research and attention, as increasing bone mass can delay the onset of osteoporosis [4]. Decreased **BMC** and **BMD** can lead to unpredictable fractures, pain, disability, significant economic burden, and reduced well-being [5].

**BMC** and **BMD** are known to be associated with various factors, including individual growth and nutritional indicators. Previous studies have investigated the individual effects of various factors, including gender, age, height, weight, hormone metabolism serum concentrations, dietary nutrients, and vitamin serum concentrations, as well as blood laboratory indicators, on **BMC** and **BMD [6–11]**. However, these studies lack a comprehensive understanding of the combined exposure of multiple vitamins, blood biochemical serum concentrations, and their association with bone health. Most studies have focused on specific bone sites and used **BMC** or **BMD** as proxies for overall skeletal health [12, 13], without evaluating the associations between **BMC**, **BMD**, and potential exposure factors across various skeletal sites. Additionally, collecting specimens with multiple vitamin exposures and different laboratory indicator serum concentrations presents significant challenges.

The **NHANES** survey conducted between 2017 and 2018 included measurements of vitamin serum concentrations and blood biochemical indicators, providing sample data for assessing bone health in relation to multiple vitamin serum concentrations and biochemical indicators. This study incorporates **BMC** and **BMD** from eight skeletal sites (skull, left arm bone, left leg bone, right arm bone, right leg bone, thoracic vertebrae, lumbar vertebrae, and pelvis) as well as whole-body measurements to represent overall skeletal health, offering a more comprehensive approach than previous studies that focused on a single skeletal site. This approach allows for a clearer identification of the specific important underlying factors influencing different skeletal sites. The study also includes individual anthropometric indicators, blood vitamin exposure serum concentrations, and selected blood biochemical indicators as potential influencing factors. Given the positively skewed distribution of **BMC** and **BMD**, the variable type for potential factors such as individual growth and development indicators, blood vitamin exposure serum concentrations, and blood biochemical indicator serum concentrations include nominal and continuous variables, with continuous data exhibiting skewed distributions.

Previous studies have used the random forest tree algorithm(**RF**) for prediction [6], and other research has shown that **RF** is a suitable ensemble learning algorithm and machine learning method, offering advantages such as independence from variable conditionality [7], high accuracy, sensitivity, and specificity compared to decision trees [7]. Furthermore, **RF** can also be used for predicting continuous variables and obtain prediction results without significant bias [8]. Therefore, **RF** is a suitable predictive method for the data in this study, allowing for the evaluation of relevant influencing factors.

This study selects participants from the **NHANES** survey conducted between 2017 and 2018, which includes assessments of bone density serum concentrations. The objective of this study is to use the vitamin exposure serum concentrations and laboratory examination indicators of the study participants as a basis to apply the **RF** method and evaluate factors related to **BMC** and **BMD**. Furthermore, the study aims to investigate whether specific factors differ in their associations with **BMC/BMD** across different skeletal sites. Assessing the association between **BMC/BMD** and the vitamin exposure serum concentrations and blood laboratory indicator serum concentrations will provide a macro-level understanding of the true associations between vitamin exposure, blood indicators, and their impact on **BMC** and **BMD**. This study aims to provide new insights into the specific vitamin exposures, blood laboratory indicator serum concentrations, and their combined effects on bone density and bone mineral content.

## 2. Materials and methods

### 2.1. Data source

**NHANES** is a cross-sectional study conducted since 1999, aimed at assessing the health and nutritional status of adults and children in the United States. **NHANES** involves interviews and physical examinations, focusing on various health and nutrition measurements, and is a major program of the National Center for Health Statistics (**NCHS**). For more detailed information, please visit the **NHANES** official website (https://www.cdc.gov/nchs/nhanes/) [9]. Considering that childhood and adolescence are characterized by rapid bone mass accumulation, this study did not include individuals under the age of 18. **NHANES** screening criteria for bone density: Participants aged 8–59 years were eligible, except for those who were pregnant (self-reported positive pregnancy test and/or self-reported use of radiographic contrast agents in the past 7 days), self-reported weight over 450 pounds or height over 6 feet 5 inches, which were excluded from Dual-energy X-ray absorptiometry (**DXA)**.

**Fig 1:**
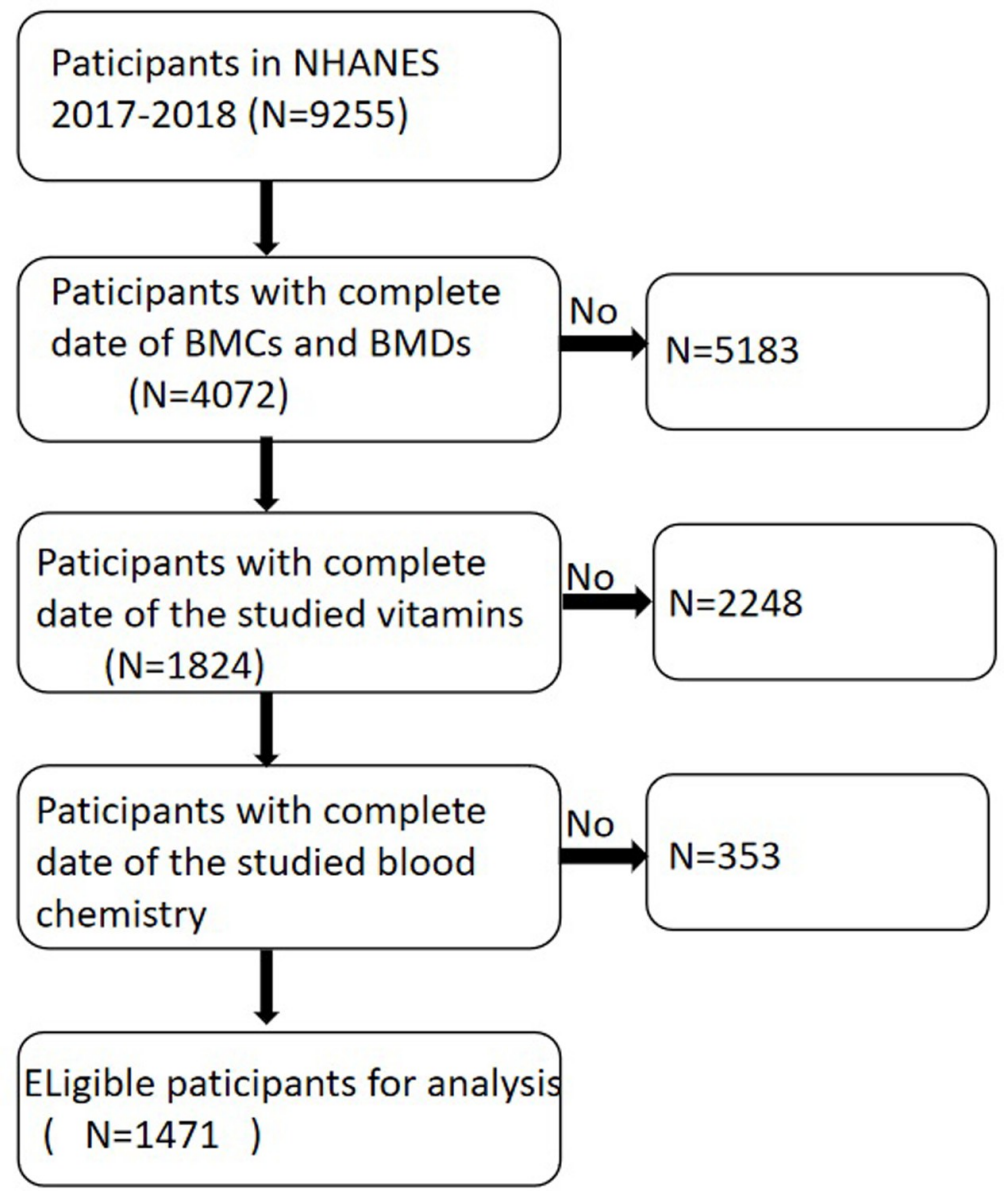
The participant exclusion flowchart.

Considering that serum serum concentrations of vitamin serum concentrations (i.e., C, D, A, and E) and blood biochemical markers were only fully measured in **NHANES** 2017–2018, this study utilized data from that cycle, including a total of 9,255 adult participants aged 19–59 years. After excluding incomplete data for **BMC** and **BMD** measurements (N = 4,072), incomplete data for vitamin serum concentrations (N = 1,824), and incomplete data for blood biochemical markers, the participant exclusion flowchart, as shown in **Fig 1**, resulted in a final sample size of 1,471.

#### 2.2.1. Ethical approval

The National Center for Health Statistics and Ethics Review Board approved the protocol for NHANES, and all participants provided written informed consent.

### 2.2 Measurement of vitamins

Collection of serum samples is conducted simultaneously with face-to-face interviews. The serum specimens are then processed, stored at -30°C, and transported to the Laboratory Science Department and CDC for analysis. Vitamins A and E are measured using high-performance liquid chromatography (HPLC) and photodiode array detection. Vitamin C is measured using isocratic HPLC, and electrochemical detection is set at 650 mV1. Serum 25-hydroxyvitamin D is measured using an equilibrium radioimmunoassay method. The NHANES Laboratory Manual provides detailed descriptions of the vitamin measurement procedures.

### 2.3 Measurement of biochemical markers

All methods were measured on the Roche Cobas 6000 (c501 module) analyzer. See Laboratory Method Files (**NHANES** Laboratory Manual for more detailed information about analyse methodologies, principles, and operating procedures. In addition, contract laboratories randomly perform repeat testing on 2% of all specimens. Based on the above, we collected the following biochemical markers: alanine aminotransferase (**ALT**)(U/L), albumin, frozen serum (g/dL), alkaline phosphatase (**ALP)**(U/L), aspartate aminotransferase (**AST**) (U/L), bicarbonate (mmol/L), blood urea nitrogen (grams/deciliter), chloride (millimole/liter), creatine phosphokinase (**CPK**) (IU/L), frozen serum creatinine (umol/L), globulin (g/dL), glucose, refrigerated serum (milligrams/deciliter), iron, refrigerated serum (micrograms/deciliter), osmolality (mmol/Kg), phosphorus, potassium, sodium, total bilirubin, total calcium, total cholesterol, total protein, triglycerides, and uric acid serum concentrations.

### 2.4 Bone density measurement

**BMC** and **BMD**: **DXA** is the most widely accepted method for measuring the body composition, partly due to its speed, ease of use, and low radiation exposure. Whole-body **DXA** scans were conducted from 2011 to 2018. **NHANES DXA** examinations provide nationally representative data on the body composition (bone and soft tissue), as well as age, sex, and race/ethnicity, to study the associations between body composition and other health conditions and risk factors such as cardiovascular disease, diabete, hypertension, and physical activity and dietary patterns. **DXA** scans provide measurements of bone and soft tissue in the whole body, arms, legs, trunk, and head. Measurements of the pelvis, left and right ribs, thoracic vertebrae, and lumbar vertebrae are also obtained. The values for the whole body and regions include: **BMC** (grams), bone area (cm2), and **BMD** (grams per square centimeter). It is worth noting that to fully illustrate the relationship between bone status, vitamin serum concentrations, and blood biochemical indices, there may be considerable differences due to different locations of the bones themselves. To avoid the occurrence of such unknown situations, we systematically evaluated the **BMC** and **BMD** of the whole body, skull, left arm bone, left leg bone, right arm bone, right leg bone, thoracic vertebrae, lumbar vertebrae, and pelvis.

### 2.5 Other variables

Sociodemographic factors (age, sex, height, weight) were obtained through face-to-face interviews. Body Mass Index (**BMI**) data were obtained through physical examinations. **BMI** is calculated by dividing weights (kg) by the square of height (m2) and classified as non-obese (<30 kg/m2) and obese (≥30 kg/m2).

### 2.6 Statistical analysis

**NHANES** Data Analysis Guidelines are retrieved from (https://wwwn.cdc.gov/nchs/nhanes/tutorials/default.aspx). In our study, if the continuous variable followed a normal distribution, it was presented as mean ± standard deviation; otherwise, it was described as median (min, max). We used a **RF** model to assess the relationships between various vitamins, blood biochemical indices, **BMC** and **BMD [9]**. To do this, we first removed several predictor variables that were highly correlated with other variables, and we retained the variables that best reflected the correlations. The variables that were removed were mainly derived variables of the correlated variables (due to the unit conversion). We then fine-tuned the model structure through the model adjustment. We explored using the grid search and ultimately selected the hyperparameters mtry = 5 and n_trees = 150 [9]. Subsequently, we constructed separate models for each body site using the selected hyperparameters. For each model, 70% of the observations were used for training, and 30% were used for testing. The training set was used to build the **RF** model, including all variables from the included samples as candidate variables, while the validation set was used to validate the model’s performance. In this study, the predicted values and actual values were evaluated using the mean absolute error (**MAE**) and root mean square error **(RMSE**) to assess the predictive performance. The coefficient of determination (**R-squared**) was used to reflect the regression fit of the prediction model. Mean accuracy was used to evaluate the relative importance of variables [10, 11]. Finally, variable importance analysis was conducted, and the variable importance in the **RF** model was estimated using the average decrease in accuracy calculated by Matlab 2016 [12–14]. This was performed for each different bone block of **BMC** and **BMD** models and summarized in the graph, showcasing the top 6 potential factors related to bone status. The statistical analysis of the aforementioned dataset was performed using IBM SPSS Statistics 22, Matlab 2016.

### 2.7 Random forest trees

**RF** is an ensemble learning method based on the Bagging approach, which combines multiple decision trees for classification and regression tasks [15]. In traditional decision trees, the selection of features for splitting is based on selecting the optimal feature from the feature set at the current node. However, in **RF**, at each node of the base decision tree, a random subset containing a particular feature k is selected from the feature set, and the optimal feature for splitting is chosen from this subset. For the input dataset, where represents the feature vector and represents the corresponding label. Let the randomly sampled dataset form the training dataset for each decision tree, and the prediction result for each decision tree, where is the number of decision trees. The prediction formula is shown in **Eq (1-1) [16]**.


$$RF(x)=\frac{1}{T}\sum_{i=1}^{T}C_{i}(x)$$ (1–1)

For randomly selected decision trees, feature importance screening, namely feature contribution assessment, Gini index or out-of-bag (OOB) error rate is commonly used as an evaluation metric. In this study, the Gini index is employed as the assessment criterion for the importance of factors **BMC** and **BMD**. The variable importance scores are denoted as VIM, and the Gini index is represented as GI. Assuming there are m features denoted as, *X*_1_,*X*_2_,…,*X*_*n*_, with K categories, and the proportion of category k in node m is *p*_*mk*_. The formula for calculating the Gini index is shown in **Eq (1-2)**.


$$GI_{m}=\sum_{k=1}^{\left|K\right|}\sum_{k^{\prime }\neq k}p_{mk}p_{mk^{\prime }}=1-\sum_{k=1}^{\left|K\right|}p_{mk}^{2}$$ (1–2)

The importance of a feature (*X*_*j*_) at node m represents the computation of the Gini index variation before and after the branching of that node (*GI*_*l*_ and *GI*_*r*_):

$$VIM_{im}^{\left(Gini\right)}=GI_{m}-GI_{l}-GI_{r}$$ (1–3)

For each tree in the ensemble, a recursive process involves random sampling of both samples and variables. If a feature (*X*_*j*_) exists in a certain node of a particular tree i among the T decision trees in the forest, the importance of that feature *X*_*C*_ is determined as follows:

$$VIM_{ij}^{\left(Gini\right)}=\sum_{m\in M}VIM_{jm}^{\left(Gini\right)}$$ (1–4)

$$VIM_{j}^{\left(Gini\right)}=\sum_{i=1}VIM_{ij}^{\left(Gini\right)}$$ (1–5)

The obtained importance scores are normalized to derive the final results [17].


$$VIM_{j}=\frac{VIM_{j}}{\sum_{i=1}^{c}VIM_{i}}$$ (1–6)

**Fig 2:**
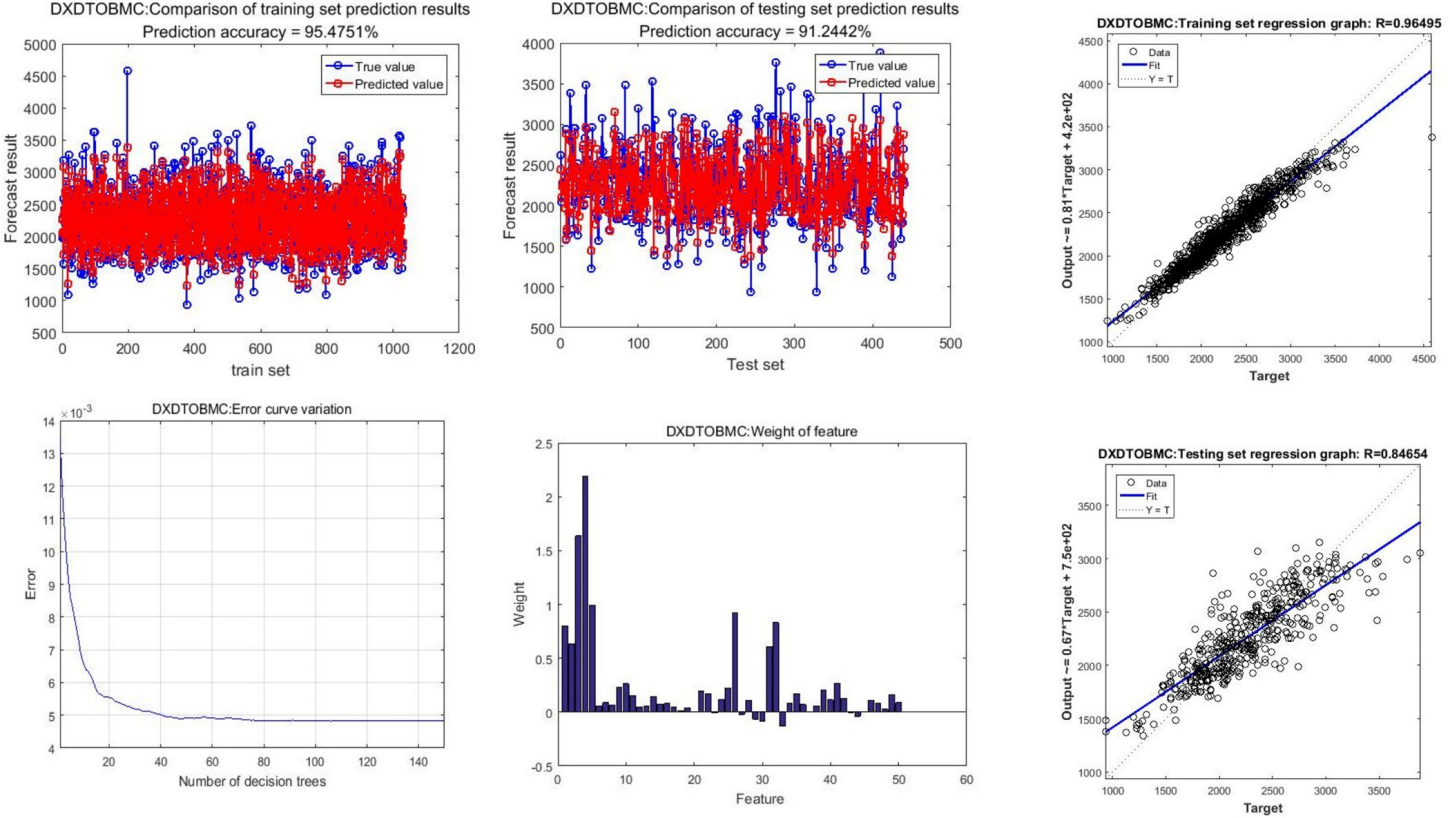
Model evaluation diagram.

The final results include the top six selections as shown in our **Table 3** and **Fig 2**, and the complete set of results can be found in S2 Table.

## 3. Results

### 3.1 Sample characteristics

This study included a total of 1,471 participants (see Table 1), with males and females accounting for 48.74% and 51.26% of the sample, respectively. The average age of the participants was 33.04 ± 14.64 years, with a height of 166.293 ± 9.49 cm, weight of 75.86 ± 20.65 kg, and **BMI** of 27.30 ± 6.68. Descriptive statistics were employed to assess the differences in **BMC** and **BMD** among different body parts. All Abbreviation of Charactors are included in **Tables 1 and 2**.

**Table 1:** Basic description of variables included in the study population.

Charactors	Abbreviation	
Sex		
Male(%)		717
Female(%)		754
Age(years)		33.04±14.64
Weight(kg)		75.86±20.65
Height(cm)		166.293±9.49
BMI		27.30±6.68
25-hydroxyvitamin D2 + D3 (nmol/L)	LBXVIDMS	65.93±29.18
25-hydroxyvitamin D2 (nmol/L)	LBXVD2MS	3.58±11.47
25-hydroxyvitamin D3 (nmol/L)	LBXVD3MS	62.41±28.8
epi-25-hydroxyvitamin D3 (nmol/L)	LBXVE3MS	4.14±2.71
alpha-carotene (μg/dL)	LBXALC	5.08±9.05
alpha-crypotoxanthin (μg/dL)	LBXARY	2.97±1.79
trans-beta-carotene (μg/dL)	LBXBEC	18.55±21.16
cis-beta-carotene (μg/dL)	LBXCBC	1.01±1.07
beta-cryptoxanthin (μg/dL)	LBXCRY	10.73±10.93
gamma-tocopherol (μg/dL)	LBXGTC	173.28±85.35
Lutein and zeaxanthin (μg/dL)	LBXLUZ	18.55±11.98
trans-lycopene (μg/dL)	LBXLYC	21.1(0.6,80.6)
Retinyl palmitate (μg/dL)	LBXRPL	1.48±1.04
Retinyl stearate (μg/dL)	LBXRST	0.54±0.19
Total Lycopene (μg/dL)	LBXLCC	41.11±18.71
Retinol (μg/dL)	LBXVIA	48.71±14.36
alpha-tocopherol (μg/dL)	LBXVIE	1086.29±373.44
Vitamin C (mg/dL)	LBXVIC	0.91(0.04,3.68)
Alanine Aminotransferase (ALT) (U/L)	LBXSATSI	21.32±15.83
Albumin, refrigerated serum (g/dL)	LBXSAL	4.2(2.9,5.2)
Alkaline Phosphatase (ALP) (U/L)	LBXSAPSI	95.07±64.27
Aspartate Aminotransferase (AST) (U/L)	LBXSASSI	21.31±11.96
Bicarbonate (mmol/L)	LBXSC3SI	25(18,33)
Blood Urea Nitrogen (g/dL)	LBXSBU	13.17±4.15
Chloride (mmol/L)	LBXSCLSI	101(91,109)
Creatine Phosphokinase (CPK) (IU/L)	LBXSCK	174.15±256.19
Creatinine, refrigerated serum (umol/L)	LBDSCRSI	71.77±27.39
Globulin (g/dL)	LBXSGB	3(1.9,5)
Glucose, refrigerated serum (mg/dL)	LBXSGL	95.53±29.11
Iron, refrigerated serum (ug/dL)	LBXSIR	88.61±39.7
Osmolality (mmol/Kg)	LBXSOSSI	278(261,299)
Phosphorus (mg/dL)	LBXSPH	3.75±0.62
Potassium (mmol/L)	LBXSKSI	4(3,5.3)
Sodium (mmol/L)	LBXSNASI	140(132,148)
Total Bilirubin (mg/dL)	LBXSTB	7.75±4.68
Total Calcium (mg/dL)	LBXSCA	9.36±0.35
Total Cholesterol, refrigerated serum (mg/dL)	LBXSCH	181.72±38.52
Total Protein (g/dL)	LBDSTPSI	72(57,88)
Triglycerides, refrigerated serum (mg/dL)	LBXSTR	128.68±95.88
Uric Acid (mg/dL)	LBXSUA	5.1(2,12)

*Unbold are the normality test passed described by mean ± standard deviation

In bold is the median (minimum, maximum) that failed the normality test

**Table 2:** Summary of whole body and different regions.

Skeletal measurements of the whole body and different regions		
Charactors	Abbreviation	
Head Bone Mineral Content (g)	DXXHEBMC	485.44(252.98,984.36)
Head Bone Mineral Density (g/cm^2)	DXXHEBMD	2.15(1.2,3.6)
Left Arm Mineral Content (g)	DXXLABMC	160.49(53.5,352.03)
Left Arm Mineral Density (g/cm^2)	DXXLABMD	0.74(0.45,1.24)
Left Leg Mineral Content (g)	DXXLLBMC	406.41 (154.41,986.06)
Left Leg Mineral Density (g/cm^2)	DXXLLBMD	1.14 (0.66,1.99)
Right Arm Mineral Content (g)	DXXRABMC	168.01 (54.17,352.03)
Right Arm Mineral Density (g/cm^2)	DXXRABMD	0.76 (0.48,1.27)
Right Leg Mineral Content (g)	DXXRLBMC	408.79 (161.81,1136.3)
Right Leg Mineral Density (g/cm^2)	DXXRLBMD	1.15(0.7,2.61)
Thoracic Spine Mineral Content (g)	DXXTSBMC	114.96 (37.51,251.08)
Thoracic Spine Mineral Density (g/cm^2)	DXXTSBMD	0.81 (0.43,1.34)
Lumbar Spine Mineral Content (g)	DXXLSBMC	51.43 (14.02,100.85)
Lumbar Spine Mineral Density (g/cm^2)	DXXLSBMD	1.01 (0.6,1.7)
Pelvis Mineral Content (g)	DXXPEBMC	234.44 (75.16,695.13)
Pelvis BMD (g/cm^2)	DXXPEBMD	1.22 (0.71,2.28)
Total Bone Mineral Content (g)	DXDTOBMC	2210.58 (934.37,4585.87)
Total Bone Mineral Density (g/cm^2)	DXDTOBMC	1.1 (0.69,1.73)

*Unbold are the normality test passed described by mean ± standard deviation

In bold is the median (minimum, maximum) that failed the normality test

### 3.2 Variable selection evaluation

**Fig 3:**
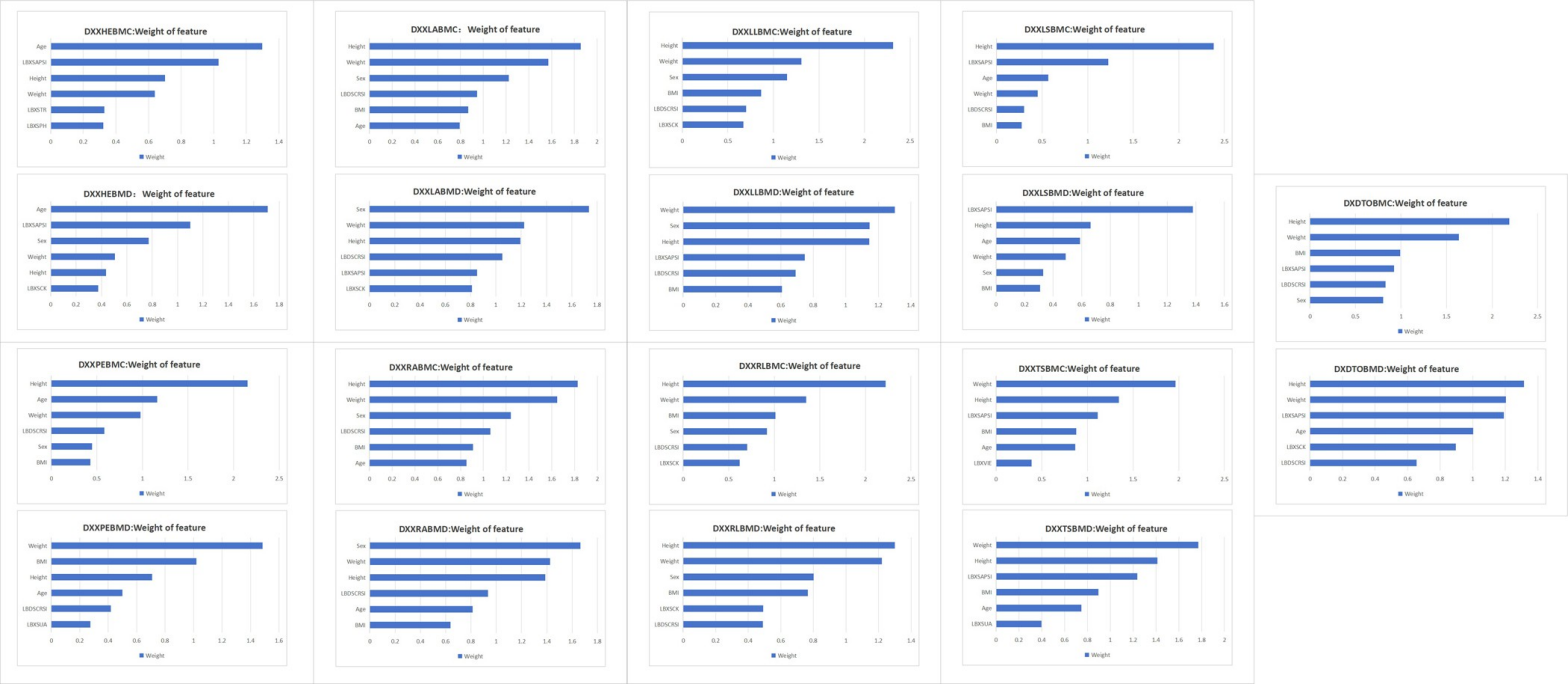
Model weight results top 6 F.

In the **RF** model, all input variables carry certain weights, and by comparing the magnitude of these weights, the relative importance of each influencing factor on the outcomes can be assessed. As the study involved a considerable number of variables, a detailed table of weights can be found in **S2 Table**. To better illustrate the important weight variables related to different bone structures, we selected the top 6 variables with high importance using the **RF** model as independent variables. **Table 3** provides a detailed listing of the top 6 variables for the importance of all input indicators regarding **BMC** and **BMD** for various bone regions, including the skull, left arm bone, left leg bone, right arm bone, right leg bone, thoracic vertebrae, lumbar vertebrae, pelvic bone, and the entire body. The most important variables, their importance rankings, and the sensitivity and specificity of the variable sets are displayed in **S1 Table**. Furthermore, weight graphs were created for the top 6 influencing factors for different bone structures, as shown in **Fig 2**. The **RF** model was evaluated for the average absolute error (**MAE**) and root mean square error (**RMSE**) between predicted and actual serum concentrations. The predictive performance was assessed using the determination coefficient **R²** to reflect the regression fit of the prediction model. The partly specific evaluation results are shown in **Fig 3**, and all model evaluation values are included in **S2 Table and S1, S2** Figs.

**Table 3:** Top 6 weighting of different variables on skeletal conditions at different sites.

DXXHEBMC	Age	LBXSAPSI	Height	Weight	LBXSTR	LBXSPH
Weight	1.2969	1.0291	0.7016	0.6382	0.328	0.3232
DXXHEBMD	Age	LBXSAPSI	Sex	Weight	Height	LBXSCK
Weight	1.7108	1.0992	0.7722	0.5067	0.4347	0.3741
DXXLABMC	Height	Weight	Sex	LBDSCRSI	BMI	Age
Weight	1.8566	1.5707	1.2245	0.9446	0.8664	0.792
DXXLABMD	Sex	Weight	Height	LBDSCRSI	LBXSAPSI	LBXSCK
Weight	1.7354	1.2234	1.1942	1.0505	0.8514	0.8114
DXXLLBMC	Height	Weight	Sex	BMI	LBDSCRSI	LBXSCK
Weight	2.3125	1.3085	1.1479	0.8648	0.6996	0.6705
DXXLLBMD	Weight	Sex	Height	LBXSAPSI	LBDSCRSI	BMI
Weight	1.3021	1.1472	1.1443	0.7478	0.6921	0.6076
DXXRABMC	Height	Weight	Sex	LBDSCRSI	BMI	Age
Weight	1.8269	1.6489	1.2401	1.0623	0.9091	0.8532
DXXRABMD	Sex	Weight	Height	LBDSCRSI	Age	BMI
Weight	1.6655	1.4244	1.3862	0.9342	0.8125	0.6377
DXXRLBMC	Height	Weight	BMI	Sex	LBDSCRSI	LBXSCK
Weight	2.2222	1.3496	1.0116	0.9177	0.7014	0.6193
DXXRLBMD	Height	Weight	Sex	BMI	LBXSCK	LBDSCRSI
Weight	1.2997	1.2199	0.8012	0.7668	0.4914	0.4903
DXXTSBMC	Weight	Height	LBXSAPSI	BMI	Age	LBXVIE
Weight	1.9661	1.3461	1.1149	0.8786	0.8678	0.3878
DXXTSBMD	Weight	Height	LBXSAPSI	BMI	Age	LBXSUA
Weight	1.7704	1.4121	1.2361	0.8957	0.7479	0.3985
DXXLSBMC	Height	LBXSAPSI	Age	Weight	LBDSCRSI	BMI
Weight	2.3821	1.2274	0.5671	0.4496	0.3006	0.2758
DXXLSBMD	LBXSAPSI	Height	Age	Weight	Sex	BMI
Weight	1.3791	0.6624	0.5878	0.4887	0.331	0.3096
DXXPEBMC	Height	Age	Weight	LBDSCRSI	Sex	BMI
Weight	2.1541	1.1608	0.9775	0.5807	0.4489	0.4272
DXXPEBMD	Weight	BMI	Height	Age	LBDSCRSI	LBXSUA
Weight	1.4854	1.0211	0.709	0.499	0.4185	0.275
DXXPEBMD	Weight	BMI	height	Age	LBDSCRSI	LBXSUA
Weight	1.4854	1.0211	0.709	0.499	0.4185	0.275
DXDTOBMD	Height	Weight	LBXSAPSI	Age	LBXSCK	LBDSCRSI
Weight	1.3159	1.2039	1.1904	1.0018	0.8954	0.6545

*Top 6 weighting (from big to small) of different variables on skeletal conditions at different sites

### 3.3 Variable selection comparison

#### 3.3.1 Skull

For instance, the top six major associated factors with **BMC** in the skull are: age, **ALP**, height, weight, **LBXSTR** (triglycerides, refrigerated serum (mg/dL)), **LBXSPH** (phosphorus (mg/dL)); the top six major associated factors with **BMD** in the skull are: age, **ALP**, sex, weight, height, creatinine phosphokinase (**CPK**) (IU/L)).

#### 3.3.2 Four limbs bone

For the left arm, the top six major associated factors with **BMC** are: height, weight, sex, **LBDSCRSI** (frozen serum creatinine (umol/L)), BMI, age; the top six major associated factors with **BMD** in the left arm are: sex, weight, height, **LBDSCRSI**, **ALP**, **CPK**; For the left leg, the top six major associated factors with **BMC** are: height, weight, sex, **BMI**, **LBDSCRSI**, **CPK**; The top six major associated factors with **BMD** in the left leg are: height, sex, weight, **ALP**, **LBDSCRSI**, **BMI**; For the right arm, the top six major associated factors with **BMC** are: height, weight, sex, **LBDSCRSI**, **BMI**, age; The top six major associated factors with **BMD** in the right arm are: sex, weight, height, **LBDSCRSI**, age, **BMI**; For the right leg, the top six major associated factors with **BMC** are: height, weight, **BMI**, sex, **LBDSCRSI**, **CPK**; The top six major associated factors with **BMD** in the right leg are: height, weight, sex, **BMI**, **CPK**, **LBDSCRSI**.

#### 3.3.3 Spine and pelvis

For the thoracic vertebrae, the top six major associated factors with **BMC** are: weight, height, **ALP**, **BMI**, age, **LBXVIE** (alpha-tocopherol (micro/dL)); the top six major associated factors with **BMD** in the thoracic vertebrae are: **ALP**, height, age, weight, sex, **LBXSUA** (uric **acid** (mg/dL)); For the lumbar vertebrae, the top six major associated factors with **BMC** are: height, **ALP**, age, weight, **LBDSCRSI**, **BMI**; The top six major associated factors with **BMD** in the lumbar vertebrae are: **ALP**, height, age, weight, sex, **BMI**; For the pelvic bone, the top six major associated factors with **BMC** are: **LBDSCRSI**, height, age, weight, sex, **BMI**; The top six major associated factors with **BMD** in the pelvic bone are: weight, **BMI**, height, age, **LBDSCRSI**, **LBXSUA**.

#### 3.3.4 The whole body

For the whole body, the top six major associated factors with **BMC** are: height, weight, **BMI**, **ALP**, **LBDSCRSI**, sex; For the whole body, the top six major associated factors with **BMD** are: height, weight, **ALP**, age, **CPK**, **LBDSCRSI**.

## 4. Discussion

This study primarily explores the associations between various vitamin exposure serum concentrations and blood biochemical indicators and **DXA**-derived **BMC** and **BMD** measurements in U.S. adults included in **NHANES** during 2017–2018. The goal is to derive the most relevant variables associated with **BMC** and **BMD** through the **RF** model. Considering the advantages of **RF** ensemble learning in mitigating overfitting and noise resistance due to the introduction of two sources of randomness (independent sampling during the construction of each decision tree and random sampling with replacement for rows and columns (attributes) of the training set [18], allowing duplicate samples), we directly include all potential influencing factors related to bone density in the machine model. Additionally, as our selected outcome variables, **BMC** and **BMD**, are continuous skewed variables, and the vitamin serum concentrations, electrolyte serum concentrations, and most laboratory biochemical indicators are also continuous skewed distributions, we opted for the adaptability of **RF**, which can handle both continuous and discrete variables without the need for normalization. The **RF** model has the advantage of automatically outputting the importance of variables, achieving a good dimensionality reduction effect. Through the aforementioned **RF** analysis, we found that the impact of blood vitamin serum concentrations on **BMC** and **BMD** may have been overestimated. In the multivariate **RF** model, we observed that blood biochemical indicators and general indicators of growth and development have greater influence weights than vitamin serum concentrations.

### 4.1. Impact of blood vitamin serum concentrations on BMC/BMD

Assessing the **BMC** and **BMD** for various bone regions, including the skull, left arm bone, left leg bone, right arm bone, right leg bone, thoracic vertebrae, lumbar vertebrae, pelvic bone, and the entire body, we did not find any vitamins to be important variables for **BMC** and **BMD**. In other words, in the jointly established model considering blood biochemical indicators, individual indicators, and blood vitamin serum concentrations, vitamin serum concentrations were not the optimal correlated variables for **BMC/BMD**. Previous studies have reported positive effects of vitamin B and vitamin D on **BMD**, while vitamin C may have a negative correlation with bone density [19–21]. Although such effects were considered, the **RF** model, which evaluates the contribution of all input variables to the model, indicated that the importance of vitamin serum concentrations is not significant under the combined influence of the body itself, the vitamin exposure, and blood biochemical indicator serum concentrations. This may explain why most vitamins have been reported to be related to bone **BMC** and **BMD**, but interventions through vitamin supplementation to regulate bone density serum concentrations are often challenging to achieve effective results [22]. One of our aims is to discover the relationship between vitamin D and various bone densities through **NHANES** 2017–2018 nutrient survey data and bone density survey data. However, we did not find that vitamin D are important influencing factors for any bone **BMC** or **BMD**.

### 4.2 Impact of blood biochemical indicator serum concentrations on BMC and BMD

Our study found that **ALP**, **CPK** and creatinine serum concentrations were identified as the most important factors related to bone health, apart from individual indicators. **ALP** was found to be an important correlated variable for **BMC** and **BMD** for all bone regions except for the left and right leg bones and the pelvic region. The substantial correlation between **ALP** and **BMC/BMD** was confirmed in our study, ALP plays a crucial role in bone formation and mineralization and is a commonly used bone turnover marker representing osteoblast activity. CPK is an enzyme involved in the metabolism of competence, mainly found in skeletal muscle, While skeletal muscle affects the bone health, thus, ALP, CPK is the most important factors related BMC and BMD. The influential mechanism of creatinine serum concentrations may be because it is related to the metabolic function, while the disorder of human metabolic function may induce the imbalance of osteoblast and osteoclast function, thus affecting the bone health, but which requires further research.

A previous study showed that an increase in serum ALP is associated with the loss of BMD and more teoporosis in females [23]. Consistent with a previous correlation study between serum T-ALP and lumbar **BMD** in young individuals, indicating a negative correlation. In clinical practice, bone alkaline phosphatase (**APK**) is closely associated with **BMD**, and further exploration is needed regard the relationship between **ALT** and **BMD [24, 25]**.

**CPK** and **LBDSCRSI** serum concentrations were also found to be significant for the limbs (left and right upper and lower limbs), possibly due to their strong correlation with the movement level of the limbs compared to other bone regions. Regarding other blood biochemical indicators, a previous multivariate regression analysis examined the relationship between the protein intake and **BMC** and **BMD**, suggesting a harmful association between protein intake and bone health. However, our evaluation of blood albumin serum concentrations did not reveal a significant correlation with bone density, possibly due to differences in the population characteristics of our study sample. One of our aims is to discover the relationship between blood calcium serum concentrations and various bone densities through **NHANES** 2017–2018 nutrient survey data and bone density survey data. However, we did not find that blood calcium serum concentrations are important influencing factors for any bone **BMC** or **BMD**.

### 4.3. Impact of general growth and development indicators on BMC and BMD

Previous studies has suggested that obesity may lead to skeletal damage [26, 27], while others have reported a positive promoting effect of fat tissue resulting from the weight gain on the bone structure. The contradictory results might not solely be due to different definitions of obesity and overweight but could also be attributed to traditional statistical methods being unable to identify the nonlinear relationship between **BMI** and **BMD**, as well as **BMC**. In contrast, our study utilized the **RF** model, taking advantage of its ability to analyze variables without conditional restrictions. Through machine learning, we effectively identified the associations between various potential influencing factors and the outcomes of **BMC** and **BMD**. Our findings indicate a significant correlation between weight, **BMI** serum concentrations, and **BMC**/**BMD**.

### 4.4. Strengths and limitations

Our study possesses several strengths. The use of the **RF** model systematically evaluated the impact weights of blood vitamin serum concentrations and blood biochemical indicators on **BMC** and **BMD** across eight different bone regions and overall bone health. This approach avoids the limitations of previous research that focused solely on vitamin serum concentrations or individual blood indicators and considered the influence on bone density in a single region. Moreover, by representing vitamin exposure serum concentrations through clear vitamin concentrations and capturing specific electrolyte and liver and kidney function serum concentrations through blood biochemical indicators, our study employed clinical outcomes such as **BMC** and **BMD** in eight differ**e**nt bone regions and the entire body. This approach provides a more comprehensive reflection of the body’s real serum concentrations and conditions.

However, our study also has some potential limitations. The use of cross-sectional survey data limits our ability to make causal inferences, necessitating evidence accumulation through large-scale prospective cohort studies and experimental research. Due to a lack of suitable data for bone metabolism(i.e. osteocalcin, cross-linking telopeptide of type I collagen) in the blood biochemical indicators, these variables were not included in the analysis. Additionally, our study focused on a population of U.S. participants, which may limit the generalizability of the results to other ethnic groups.

## 5. Conclusion

Using the **RF** model and data from **NHANES** 2017–2018 for adults aged 19–59 years in the United States, we explored the relationship between exposure to multiple vitamins, body serum concentrations of biochemistry and **BMC** and **BMD**. Under **RF** dimension reduction and comparison selection, the effect of vitamin serum concentrations on human bone density was not significant. **ALP**, **CPK** and creatinine serum concentrations were identified as the most important factors related to bone status, and body development indicators were also important variables related to **BMC** and **BMD**, but vitamin D and blood calcium serum concentrations may not affect the serum concentrations of **BMC** and **BMD**. We suggest using statistical methods such as **RF** to comprehensively evaluate the effects of vitamin content and blood biochemistry serum concentrations in adults on **BMC** and **BMD.** This open up a new direction for further exploring the evidence between multiple vitamin exposure, blood indicator exposure, and **BMC**/**BMD**.

## Supporting information

S1 Table Summary of the model evaluation table. (DOCX)

S2 Table All weighting of different variables on skeletal conditions at different sites. (XLSX)

S1 Fig Model evaluation diagram. (PNG)

S2 Fig Evaluation plot of the fitting results. (PNG)

S1 File (XLS)
