# Correction: Relationships between multivitamins, blood biochemistry markers, and BMC and BMD based on RF: A cross-sectional and population-based study of NHANES, 2017–2018

**DOI:** 10.1371/journal.pone.0321774

**Published:** 2025-04-01

**Authors:** Lijuan Xu, Mengqi Wu, Ying Zhang, Hongsheng Kun, Jiangbao Xu

There is an error in affiliation 1 for author Lijuan Xu. The correct affiliation 1 is: Jiangshan People’s Hospital, Jiangshan, Zhejiang Province, China.
