# Supplementary material for: Relationships between multivitamins, blood biochemistry markers, and BMC and BMD based on RF: A cross-sectional and population-based study of NHANES, 2017–2018
Source: PLoS One. 2025 Jan 29;20(1):e0309524. doi: 10.1371/journal.pone.0309524 (PMC11778711; doi:10.1371/journal.pone.0309524)
Supplement: S1 Table — (DOCX) [file pone.0309524.s001.docx]

| Training sets | SSE | MAE | MSE | RMSE | MAPE | Accuracy | R^2^ |
| --- | --- | --- | --- | --- | --- | --- | --- |
| DXXHEBMC | 1876388.58 | 33573.51 | 1876388.58 | 1369.81 | 6.84% | 93.16% | 0.94 |
| DXXHEBMD | 26.59 | 123.89 | 26.59 | 5.16 | 5.60% | 94.40% | 0.94 |
| DXXLABMC | 149350.44 | 9017.55 | 149350.44 | 386.46 | 5.43% | 94.57% | 0.97 |
| DXXLABMD | 1.02 | 23.66 | 1.02 | 1.01 | 3.07% | 96.93% | 0.96 |
| DXXLLBMC | 774949.46 | 20494.96 | 774949.46 | 880.31 | 4.79% | 95.21% | 0.97 |
| DXXLLBMD | 3.19 | 41.58 | 3.19 | 1.79 | 3.49% | 96.51% | 0.94 |
| DXXRABMC | 156584.42 | 9443.56 | 156584.42 | 395.71 | 5.36% | 94.64% | 0.97 |
| DXXRABMD | 1.14 | 24.53 | 1.14 | 1.07 | 3.17% | 96.83% | 0.96 |
| DXXRLBMC | 915196.92 | 20148.52 | 915196.92 | 956.66 | 4.65% | 95.35% | 0.96 |
| DXXRLBMD | 3.84 | 3.84 | 3.84 | 1.96 | 3.25% | 3.25% | 0.93 |
| DXXTSBMC | 110218.66 | 7797.96 | 110218.66 | 331.99 | 6.75% | 93.25% | 0.95 |
| DXXTSBMD | 106996.34 | 7888.1 | 106996.34 | 327.1 | 6.89% | 93.11% | 0.95 |
| DXXLSBMC | 32555.95 | 4407.67 | 32555.95 | 180.43 | 8.64% | 91.36% | 0.94 |
| DXXLSBMD | 5.46 | 56.66 | 5.46 | 2.34 | 5.42% | 94.58% | 0.94 |
| DXXPEBMC | 767556.19 | 20767.88 | 767556.19 | 876.1 | 8.46% | 91.54% | 0.95 |
| DXXPEBMD | 5.69 | 57.15 | 5.69 | 2.39 | 4.51% | 95.49% | 0.94 |
| DXDTOBMC | 18987927.2 | 103445.57 | 103445.57 | 103445.57 | 4.52% | 95.48% | 0.96 |
| DXDTOBMD | 2.33 | 36.17 | 2.33 | 1.53 | 3.21% | 96.79% | 0.94 |
| Testing sets | SSE | MAE | MSE | RMSE | MAPE | Accuracy | R^2^ |
| DXXHEBMC | 3341803.67 | 28910.64 | 3341803.67 | 1828.06 | 13.42% | 86.58% | 0.54 |
| DXXHEBMD | 39.78 | 104.13 | 39.78 | 6.31 | 11.28% | 88.72% | 0.6 |
| DXXLABMC | 217267.12 | 7357.76 | 217267.12 | 466.12 | 10.57% | 89.43% | 0.89 |
| DXXLABMD | 1.81 | 20.88 | 1.81 | 1.34 | 6.21% | 93.79% | 0.81 |
| DXXLLBMC | 1192542.06 | 16606.77 | 1192542.06 | 1092.04 | 9.09% | 90.91% | 0.88 |
| DXXLLBMD | 3.81 | 31.81 | 3.81 | 1.95 | 6.34% | 93.66% | 0.74 |
| DXXRABMC | 196068.54 | 7254.63 | 196068.54 | 442.8 | 10.30% | 89.70% | 0.9 |
| DXXRABMD | 1.5 | 19.75 | 1.5 | 1.5 | 6.00% | 94.00% | 0.8 |
| DXXRLBMC | 1317257.6 | 17023.68 | 1317257.6 | 1147.72 | 8.93% | 91.07% | 0.87 |
| DXXRLBMD | 4.54 | 35.3 | 4.54 | 2.13 | 6.94% | 93.06% | 0.72 |
| DXXTSBMC | 151824.03 | 6470.43 | 151824.03 | 389.65 | 13.20% | 86.80% | 0.77 |
| DXXTSBMD | 157596.73 | 6238.44 | 157596.73 | 396.98 | 12.03% | 87.97% | 0.76 |
| DXXLSBMC | 46042.07 | 3517.94 | 46042.07 | 214.57 | 16.37% | 83.63% | 0.59 |
| DXXLSBMD | 8.33 | 46.8 | 8.33 | 2.89 | 10.76% | 89.24% | 0.45 |
| DXXPEBMC | 1148543.49 | 16442.43 | 16442.43 | 1071.7 | 15.27% | 84.73% | 0.72 |
| DXXPEBMD | 8.48 | 47.5 | 8.48 | 2.91 | 8.79% | 91.21% | 0.55 |
| DXDTOBMC | 28903683.93 | 85318.5 | 28903683.93 | 5376.21 | 8.76% | 91.24% | 0.85 |
| DXDTOBMD | 3.49 | 30.53 | 3.49 | 1.87 | 6.33% | 93.67% | 0.67 |
