# Supplementary figures and images for: Relationships between multivitamins, blood biochemistry markers, and BMC and BMD based on RF: A cross-sectional and population-based study of NHANES, 2017–2018
Source: PLoS One. 2025 Jan 29;20(1):e0309524. doi: 10.1371/journal.pone.0309524 (PMC11778711; doi:10.1371/journal.pone.0309524)

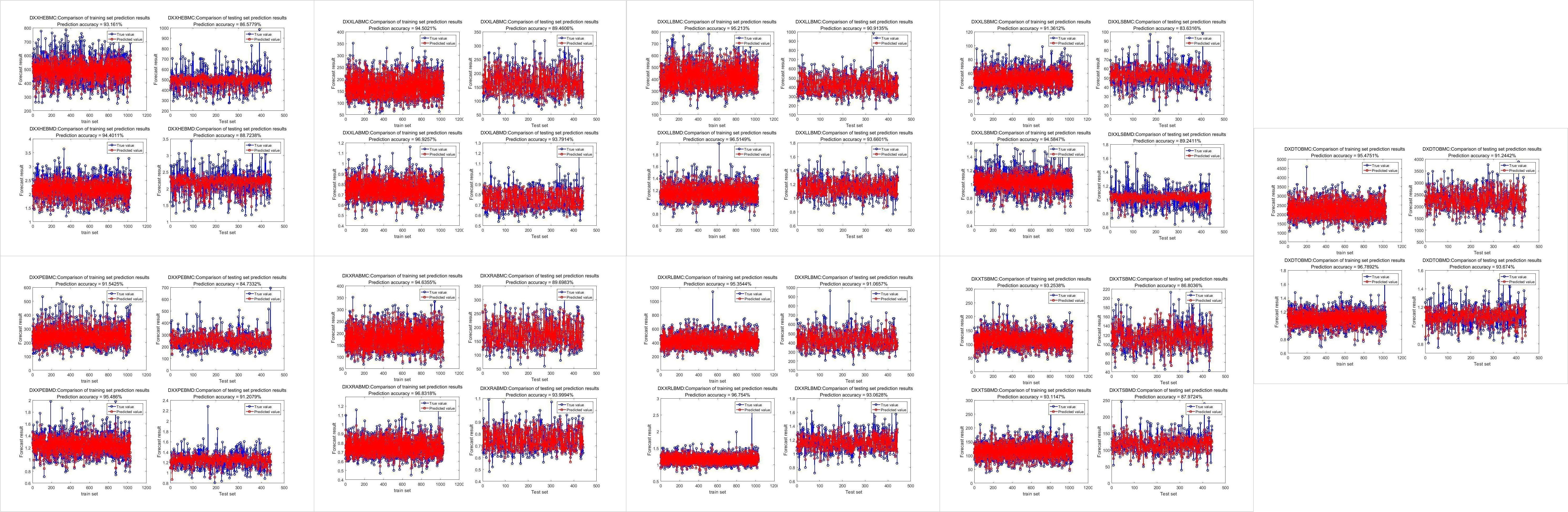

Supplement: S1 Fig — (PNG) [file pone.0309524.s003.png]

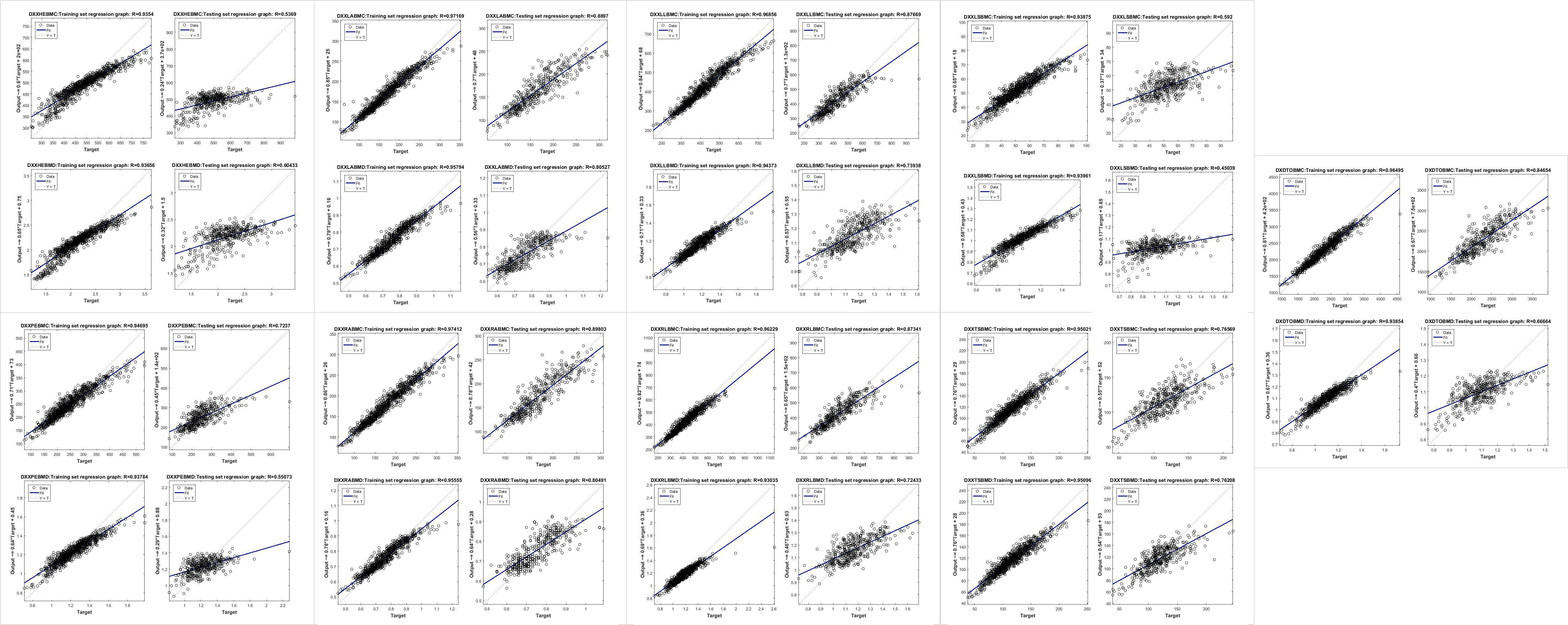

Supplement: S2 Fig — (PNG) [file pone.0309524.s004.png]
